# Native Aortic Valve Endocarditis Secondary to Gastric Antral Vascular Ectasia Manipulation During Endoscopic Argon Plasma Coagulation

**DOI:** 10.7759/cureus.53930

**Published:** 2024-02-09

**Authors:** Ranjit Nair, Jude Elsaygh, Anas Zaher, Michael Fragner, Gila Perk

**Affiliations:** 1 Internal Medicine, New York-Presbyterian Brooklyn Methodist Hospital, New York City, USA; 2 Cardiology, New York-Presbyterian Brooklyn Methodist Hospital, New York City, USA

**Keywords:** transesophageal echocardiogram, argon plasma coagulation, gastric antral vascular ectasia, murmur, infective endocarditis

## Abstract

Gastric antral vascular ectasia (GAVE) is an uncommon cause of upper gastrointestinal (GI) bleeds. Due to the high vascularity of the region, transient bacteremia due to manipulation of the GI tract can very rarely cause the translocation of bacteria. We present a rare case in which endoscopic manipulation to treat GAVE led to native valve infective endocarditis (IE). Our patient had a prior history of GAVE and presented with worsening dizziness and shortness of breath (SOB). After an esophagogastroduodenoscopy (EGD) and subsequent argon plasma coagulation (APC) for active preantral bleeding, the patient was noted to have repeated fevers, a new cardiac murmur, and positive blood cultures for *Staphylococcus epidermidis*, leading to a diagnosis of native infective endocarditis. With high clinical suspicion and early recognition of a new cardiac murmur, a transesophageal echocardiogram (TEE) was key in identifying vegetation. This case highlights the importance of combining history, a physical exam, and diagnostic lab tests and imaging to identify endocarditis. Management included two months of intravenous (IV) vancomycin and repeat TEE for close monitoring of vegetation improvement.

## Introduction

Gastric antral vascular ectasia (GAVE) is responsible for about 4% of non-variceal upper gastrointestinal (GI) bleeds [1]. In the disorder, the antral stomach lining can bleed in several different locations due to dilated capillaries in the lamina propria and fibrin thrombi [2]. Gastric antral vascular ectasia (GAVE) is considered an acquired disease where mechanical stress for any reason within the stomach, such as strong peristaltic waves, is thought to contribute towards its pathogenesis, leading to vascular ectasia [2]. Treatment modalities may include surgical antrectomy, pharmacological therapy with hormones or somatostatin analogues, or endoscopic procedures such as argon plasma coagulation (APC). Argon plasma coagulation (APC) has reportedly been shown to be safer and similar in effectiveness to surgery [2]. This procedure uses argon plasma to transduce electrical energy to tissue and to coagulate superficial, bleeding vessels within the stomach wall. Adverse effects can include antral ulcers, gastric polyps, Mallory-Weiss syndrome, scarring of the stomach lining, and even sepsis [3]. However, bacteremia causing native aortic valve endocarditis after APC in the treatment of GAVE’s disease has not yet been described and is exceedingly rare.

## Case presentation

**Figure 1:**
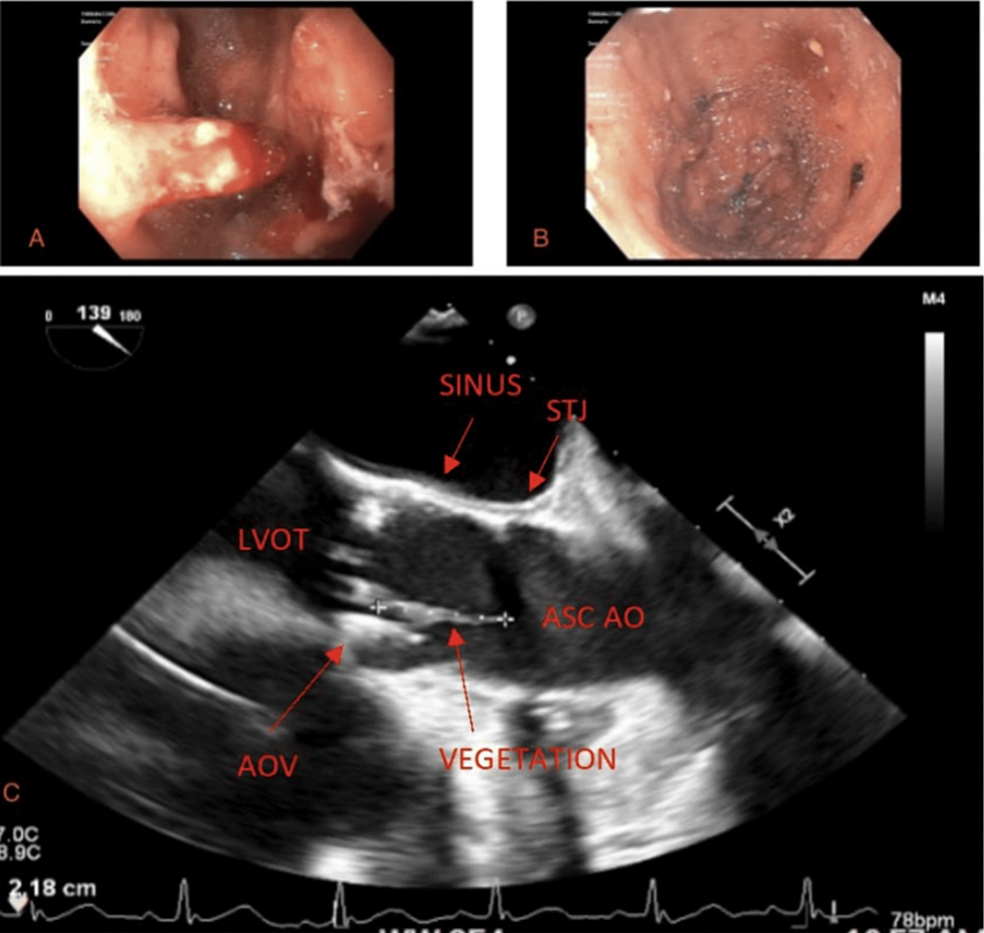
Endoscopic stomach views before and after argon plasma coagulation and subsequent vegetation on the aortic valve 1A) Pre-pyloric region with active bleeding from gastric antral vascular ectasia 1B) gastric antral vascular ectasia post-argon plasma coagulation. 1C) Transesophageal echocardiogram, mid-esophageal long-axis view at the level of the aortic valve showing a 2-centimeter vegetation coming off the right coronary cusp of the aortic valve.

**Figure 2:**
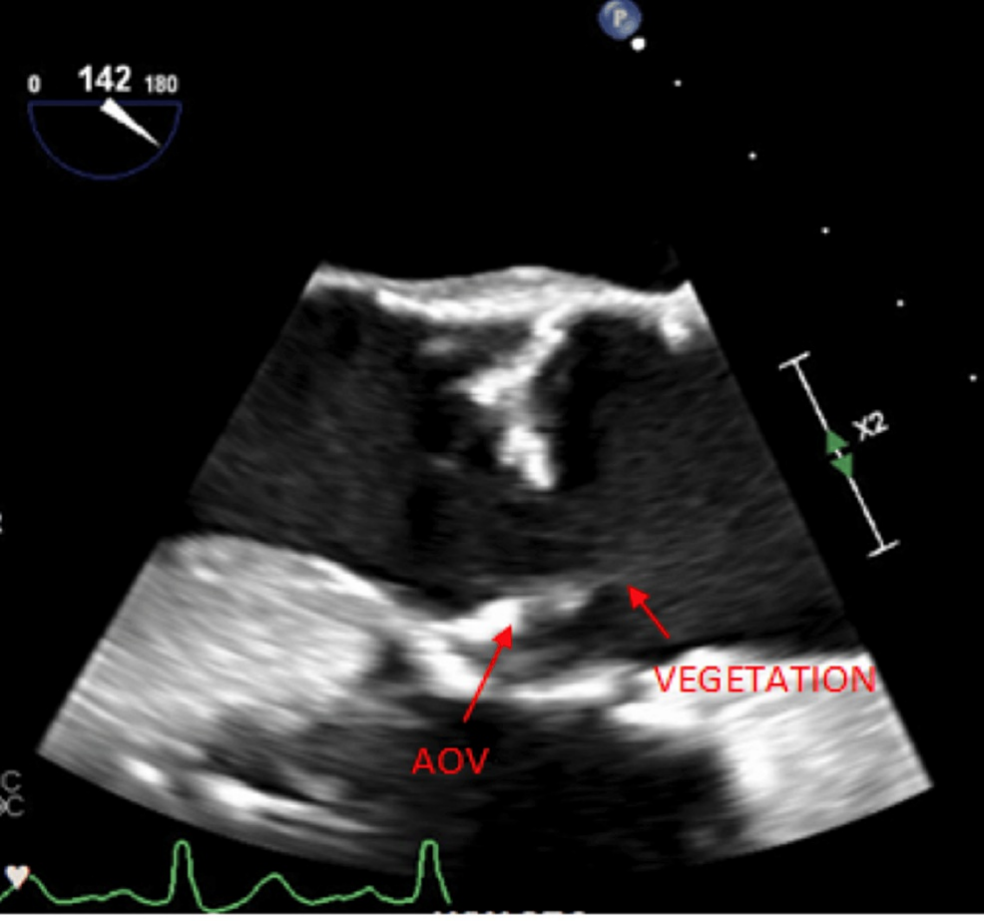
Remnant of aortic valve vegetation after antibiotics Transesophageal echocardiogram post-IV antibiotics showing minimal strands of vegetation. AOV: aortic valve; LVOT: left ventricular outflow tract; SINUS: sinus of Valsalva; STJ: sino tubular junction; ASC AO: ascending aorta

In our case report, a 69-year-old male with a history of atrial fibrillation status post-Watchman device and multiple upper GI bleeds in the setting of GAVE’s disease presented to the emergency department for shortness of breath, dizziness, and melanotic stools. His lab reports were significant for acute blood loss anemia, as noted by a 3-unit hemoglobin drop from baseline. The patient underwent an endoscopy, which revealed GAVE with active bleeding in the prepyloric region (Figure 1A). The patient underwent argon plasma coagulation to control the bleeding with success (Figure 1B). However, on post-operative day two, the patient spiked a fever to 38°C. Two sets of blood cultures were positive for gram (+) cocci in clusters, which later grew *Staphylococcus epidermidis*. A new systolic murmur was also appreciated on subsequent physical examinations. A transthoracic echocardiogram (TTE) revealed a heavily calcified aortic valve with severe aortic stenosis but no regurgitation. A TTE from two months prior showed moderate aortic stenosis and no regurgitation. A transesophageal echocardiogram (TEE) was conducted to follow up on the TTE findings and showed a two-centimeter mobile vegetation on the aortic valve (Figure 1C). The patient was subsequently treated for infective endocarditis (IE) with a six-week course of intravenous vancomycin (1.25 gm every 12 hours). A repeat TEE showed a thickened and irregular-appearing aortic valve, but only with a thin strand of remnant from the vegetation and no valve destruction (Figure 2).

## Discussion

Argon photocoagulation during endoscopic manipulation has been a mainstay of GAVE treatment and active bleeding control. Unfortunately, the long-term efficacy and complications of therapy have not been well defined. Recurrent bleeding rates can be as high as 35%-78.9% and further sessions of APC can expose patients to other complications [4]. Infective endocarditis caused by the subsequent bacteremia, induced by endoscopic manipulation and photocoagulation, is a highly rare complication that has not been previously discussed. Especially in older populations, a lower threshold for testing is necessary when there is suspicion of endocarditis, as the presentation can be atypical with nonspecific constitutional signs and symptoms. This can include fatigue, lethargy, delirium, or weight loss [5]. Fevers can even be absent [5]. In our patient, two febrile episodes had prompted the acquisition of blood cultures more than 12 hours apart, with both resulting positive for *Staphylococcus epidermidis*. In patients with degenerative valvular thickening or even a severely calcified valve, like the patient in our case, TTE findings of endocarditis can be difficult to interpret, necessitating a TEE to further assess the diagnosis [6]. In this case, the diagnosis of native valve infective endocarditis was made based on a constellation of symptoms and signs, which included recurrent fevers, a new cardiac murmur, positive blood cultures, and aortic valve vegetation seen on TEE.

A few limitations of this case can be discussed. For example, there is a lack of definitive evidence that endoscopic manipulation was the sole cause of valvular endocarditis. Blood cultures were first collected two days after the endoscopy and APC because the patient only developed a low-grade fever at that time. Although the procedure, which resulted in the manipulation of highly vascularized tissue, likely created a nidus for valvular infection, it is not improbable that other factors during hospitalization or outside of the hospital could have done so as well. In this patient, a severely calcified aortic valve significantly increases the risk for infective endocarditis in general, despite the mode of bacterial entry [7]. However, looking at the aforementioned timeline, endoscopic manipulation with APC of a highly vascularized structure within the stomach appears to be the most likely culprit. In addition, while endocarditis could have worsened a systolic murmur, prior TTE conducted two months before this hospitalization noted moderate aortic stenosis with moderate calcifications. A “new” cardiac murmur was included as part of the workup and criteria to diagnose the patient’s endocarditis when it is entirely possible this finding was present before the infection but missed.

In our patient, a six-week course of vancomycin resulted in significant vegetation resolution from the aortic valve. However, during future hospital courses, the patient continued to undergo several endoscopies and the use of hemospray, banding, and APC to control gastric bleeds due to GAVE and gastric angiodysplasias. At this time, a decision to perform a transaortic valve replacement in the setting of severe aortic stenosis is pending, given the patient’s ongoing medical dilemmas.

## Conclusions

Patients with recent manipulation of GAVE, febrile episodes, bacteremia with typical organisms for IE, and a new valvular murmur should raise suspicion for IE. Clinicians need to have a lower threshold for further diagnostic testing, such as repeat TTE and TEE, in older patients with a previous history of damaged or calcified cardiac valves.
